# Design and Synthesis of Anti-Cancer Chimera Molecules Based on Marine Natural Products

**DOI:** 10.3390/md17090500

**Published:** 2019-08-27

**Authors:** Min Woo Ha, Bo Reum Song, Hye Jin Chung, Seung-Mann Paek

**Affiliations:** College of Pharmacy and Research Institute of Pharmaceutical Sciences, Gyeongsang National University, Jinju Daero 501, Jinju 52828, Gyeongnam, Korea

**Keywords:** chimera, chemical conjugation, marine natural products, anticancer agent, hybridization

## Abstract

In this paper, the chemical conjugation of marine natural products with other bioactive molecules for developing an advanced anti-cancer agent is described. Structural complexity and the extraordinary biological features of marine natural products have led to tremendous research in isolation, structural elucidation, synthesis, and pharmacological evaluation. In addition, this basic scientific achievement has made it possible to hybridize two or more biologically important skeletons into a single compound. The hybridization strategy has been used to identify further opportunities to overcome certain limitations, such as structural complexity, scarcity problems, poor solubility, severe toxicity, and weak potency of marine natural products for advanced development in drug discovery. Further, well-designed marine chimera molecules can function as a platform for target discovery or degradation. In this review, the design, synthesis, and biological evaluation of recent marine chimera molecules are presented.

## 1. Introduction

The struggle for existence in a natural environment occasionally makes a species develop its own unique weapons such as speed, power, or even toxins. As these toxins possess biologically potent activity and unique modes of actions, these natural products have been regarded as a robust platform for further medicinal research [1,2]. Thus far, marine natural products have been particularly highlighted for their extraordinary bioactivity under highly diluted conditions [3,4,5]. Therefore, it is plausible to utilize marine natural products as a hit or lead compound in drug discovery and its further development [6,7]. 

**Figure 1:**
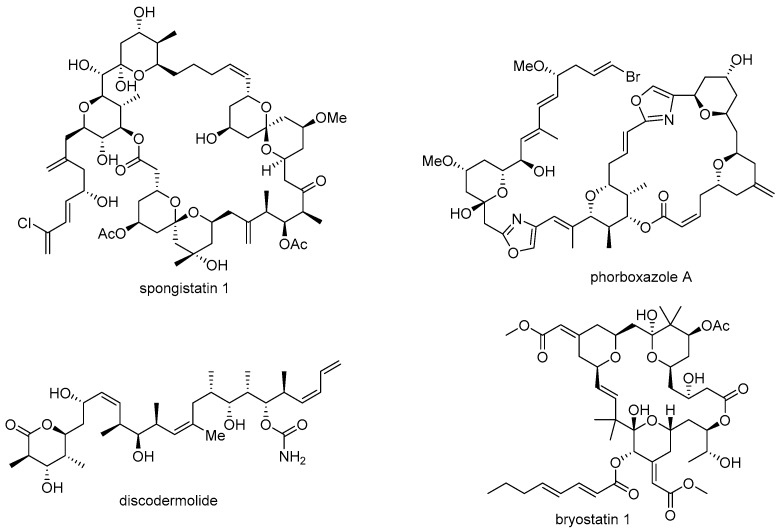
Structure of certain bioactive marine natural products.

Although marine natural products could have the powerful potential for drug discovery, there are also a few obstacles associated with them. First, it is rather difficult to secure a sufficient amount of these products for further study [8]. In most cases, the medicinal study of natural products requires a substantial number of test samples for elucidation of target protein/receptor and following signaling pathway. However, harsh conditions, difficulty in access, and scarcity of the target organism are hindrances to large scalable synthesis of important marine natural products, such as spongistatin 1 (13.8 mg from 400 kg of marine sponge) [9], phorboxazole A (95 mg from 236 g of *Phorbas* sp.) [10], discodermolide (7 mg from 434 g of *Discodermia dissoluta*) [11], bryostatin 1 (0.9~1.8 g from 10,000 gallons of *Bugula neritina*), [12,13] etc. The unmet needs from natural resources occasionally leads to samples being obtained via chemical synthesis, and it usually remains difficult to satisfy substantial supply requirements [14,15,16] (Figure 1).

**Figure 2:**
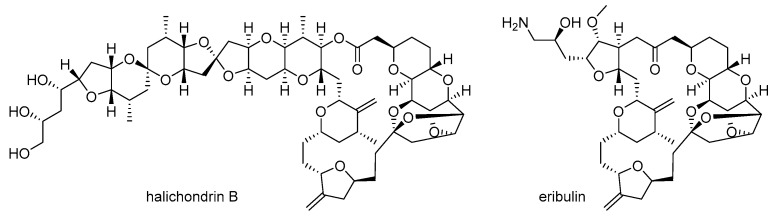
Halichondrin B [21] and its synthetic analog eribulin [22].

Second, structural complexity is another hurdle. The highly complex structure of marine natural products frequently makes it very difficult to modify or synthesize them on a large scale [17,18]. In order to improve the biological activity of these products, both chemical modification and related structure-activity relationship (SAR) study of marine natural products are necessary [19]. However, their highly complex structure hampers efficient modifications and any subsequent systematic research [20]. In order to address this structural complexity, a more simplified analog of marine natural products can be a breakthrough in drug discovery, as evident in the case of halichondrin B [21] and its simplified analog eribulin [22] (Figure 2). Although halichondrin B possesses extraordinary cytotoxicity against B-16 melanoma cells (IC_50_ 0.093 ng/mL) and other tumor cell lines, its structure is too complex to be an advanced drug or drug candidate for related cancer therapy. Consequently, the truncated ketone analog eribulin of halichondrin B was developed through an entirely synthetic approach [23] and approved for treatment of metastatic breast cancer as a mesylate salt.

The third hurdle is biological activity and its selectivity. Since marine natural products are derived from marine organisms and not humans, their application to the human body may trigger undesired biological processes [24]. In order to solve this selectivity issue, various analogs are made and tested [25,26]. In addition, systematic study for mechanism of action or ligand-target binding usually follows valuable analogs [27]. Nonetheless, marine natural products continue to serve as a versatile starting point for drug discovery because of their unique structural framework and biological activity. Simultaneously, the strategy for the efficient modification of this complex molecule has also been evolving.

**Figure 3:**
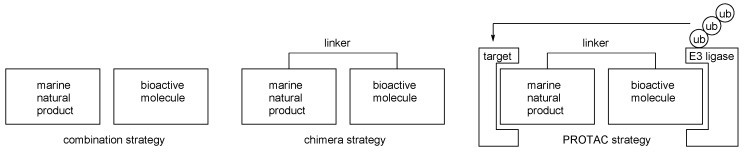
Comparison of the combination and chimera strategies.

Molecular hybridization can be a good strategy for advanced marine natural products [28,29,30,31]. Certain drawbacks of marine natural products such as structural complexity or non-selective biological activities were overcome through a combination with other bioactive molecules. This combination strategy was evolved to develop chimera molecules, pursuing not merely a synergistic sequence but a systematically operating biological sequence. For example, the hybridization of a ligand and ubiquitin recruiting probe enables the degradation of target biomolecules via cellular sequence, such as ubiquitination and following the proteasome pathway. This proteolysis targeting chimera (PROTAC) approach showcases a well-designed chimera strategy from natural products [32,33,34,35,36,37]. (Figure 3) In addition, biotinylation of active ligand provides affinity column chromatography to enable target protein isolation [38]. In addition to these strategies, an improvement of basic activity can be anticipated. In this regard, advanced chimeric molecules from marine natural products with anticancer activity is reviewed here.

## 2. Results

### 2.1. Conjugation with Other Active Molecules

The macrosphelide (MS) family [39], comprising 13 natural isomers, are 15- or 16-membered macrolide antitumor agents. They are derived from marine sponge *Periconia byssoides* [40] (MSC **1**, MSE-H, and MSL) and soil fungi *Microsphaeropsis* sp. FO-5050 [41] (MSA-D, MSJ, and MSK) and show cell adhesion inhibitory activity or immunosuppressive activity [42]. In addition, a derivatization of macrosphelide skeleton based on MSA **2** or MSE **3** enables the activation of apoptosis in human lymphoma U937 cells, although their activity is slightly weak [43]. In order to increase this apoptosis-inducing activity in cancer cells, preparation of a chimera compound with another 16-membered anticancer agent, epothilone **4**—which shows not only tubulin-disrupting profiles and anticancer activity, similar to the paclitaxel, but also an apoptosis-inducing property in cancer cells to inhibit tumor cell growth—was pursued [44]. Based on their similar structural features and biological properties, hybridization of MS and epothilone **4** was performed, pursuing an advanced anticancer agent.

**Scheme 1:**
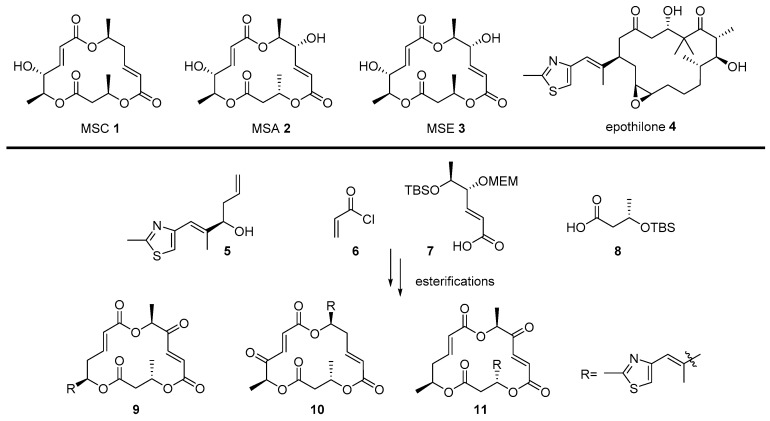
Hybridization of macrosphelide (MS) skeleton and epothilone side chain [44].

Scheme 1 illustrates the synthetic plan of the MS-epothilone chimera. Known intermediate **5 [45]** for total synthesis of epothilone was prepared and linked to fragments **6**, **7**, [42] and **8** of MS via iterative esterification and deprotection of secondary alcohol moiety. For an elucidation of the structure-activity relationship, a thiazole side chain of epothilone was introduced in each CH_3_ group in the MS skeleton to provide the desired MS-epothilone chimera **9**–**11**.

Screening of chimeras **9**–**11** exhibited an increase in their desired potencies, which is attributed to this hybridization strategy. In particular, chimera 9 showed more potent apoptosis-inducing activity in the U937 cell line as compared to other analogs or parent compounds MSA **2** or MSE **3**. It is noteworthy that other chimeras also showed an increased apoptotic property, while parent MSA **2** or MSE **3** did not have the same activity at the same concentration (1 μM, 12 h incubation). Further, successful hybridization of MS with epothilone **4** presents that the chimeric molecule strategy can be a powerful solution to the limitation of marine natural products themselves.

**Scheme 2:**
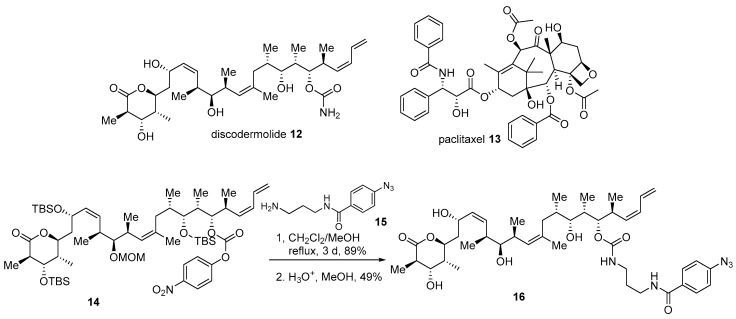
Hybridization of discodermolide **12** with side chain moiety of paclitaxel **13** [47].

The complex structure of marine products can hamper efficient design or synthesis of advanced chimera molecules. In such cases, in silico study may offer another solution. Discodermolide **12**, a marine polyketide product from *Discodermia dissolute*, possesses potent antiproliferative activity against various human cancer cell lines [11]. Mechanically, it stabilizes microtubules and finally arrests cells in the mitosis status, just as paclitaxel does [46]. However, unlike paclitaxel **13**, a previous SAR and docking study of discodermolide at the paclitaxel binding site of tubulin revealed that free carbamate of discodermolide could be modified [47]. Based on the following study, conjugated diene in discodermolide resides in an aromatic pocket of tubulin. Moreover, the attachment of a simple aromatic group did not decrease its own anticancer activity. With this early study, the introduction of an aromatic side chain to discodermolide was pursued [47]. Synthetic intermediate **14** was coupled with various amine side chains, such as **15** with photolabile functionality, to finally produce carbamate **16** in good yield (Scheme 2). It was gratifying to note that the biological evaluation of this hybrid **16** showed an improved antiproliferative profile against human cancer cell lines. When it was treated to the lung cancer cell line A549, inhibition of cell growth at IC_50_ 1.21 ± 0.35 nM was demonstrated, while discodermolide **12** showed a value of IC_50_ 9.34 ± 0.56 nM (paclitaxel IC_50_ 3.14 ± 0.09 nM). This pharmacological advance implies that the hybridization strategy based on the docking study could be another option for structurally complex molecules.

**Scheme 3:**
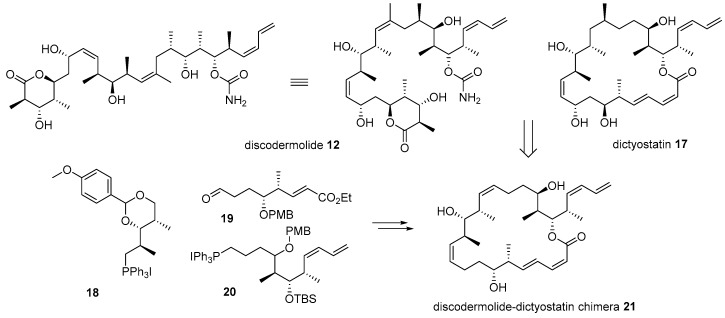
Structural comparison and hybridization of discodermolide **12** with dictyostatin **17** [48].

Further, hybridization of discodermolide **12** and dictyostatin **17** was also performed based on their similar structural features and anticancer activities, as depicted in Scheme 3 [48]. Dictyostatin **17** is a 22-membered macrolide natural product from the Indian [49] or Caribbean ocean sponge [50]. Dictyostatin has also garnered substantial attention due to its extraordinary antiproliferative effect (ED_50_ 0.38 nM for P388 leukemia cell) [51]. A comparison of discodermolide and dictyostatin makes it plausible to say that they have similar backbones and substituents, except for the 22-membered lactone of dictyostatin. Based on this observation, hybridization of these two powerful anticancer natural products was performed [48]. During the synthesis and SAR study of discodermoilde **12**, a practical synthetic route to it was developed and applied to chimera preparation. *Para*-methoxybenzyl (PMP)-acetal **18** was coupled with aldehyde **19** using Wittig olefination to construct pivotal Z-alkene of target chimera. After some functional group interconversion and another Wittig olefination with diene **20**, discodermolide-dictyostatin chimera **21** was efficiently prepared. In addition, the biological activity of the chimera was also examined. Although chimera **21** possesses a relatively simple structure compared to discodermolide **12**, it displayed one-third the potency of discodermolide in a displacement test using [^3^H]-paclitaxel bound to microtubules. This simplified structure with moderate activity might open new possibilities for further development of related natural products.

Since great cytotoxicity for solid tumors was observed using *Psammocinia* extracts from the waters of Papua New Guinea [52], significant effort has been made to isolate active ingredients produced from moderated cytotoxic marine natural products, such as cyclocinamide A [53], swinholide A [54], and furanosesterpenes [55]. However, these compounds did not explain the extraordinary cytotoxic properties of crude extract. The active ingredient was re-examined only in the early 2000s. Finally, highly anti-proliferative marine natural product psymberin **22** was isolated [52,56], but its absolute configuration and C_4_-stereochemistry were not confirmed. An additional research program elucidated its mysterious structure through total synthesis by the De Brabander group [57]. 

**Scheme 4:**
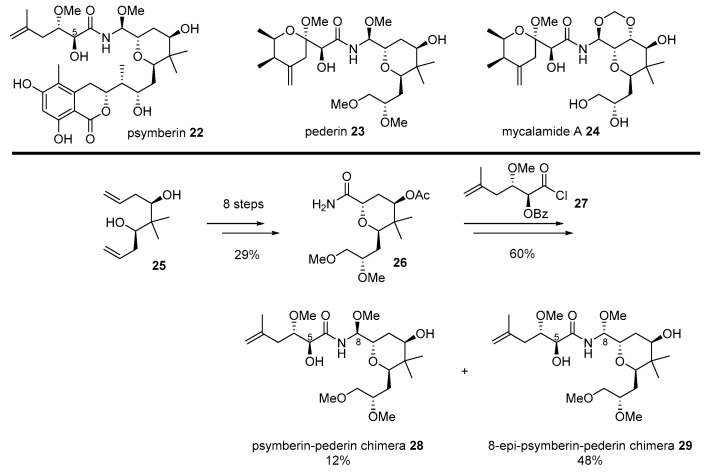
Hybridization of psymberin **22** with pederin **23** [59].

After confirmation of the correct structure, psymberin **22** was compared to classic natural compounds pederin **23** from rove beetles or mycalamide A **24** from marine invertibrates [58]. Although their structure possesses unique aminal-amide and trans-substituted tetrahydropyran skeletons, psymberin **22** has a characteristic dihydroisocoumarin moiety, while pederin/mycalamide A has another tetrahydropyran with the exomethylene group. As pederin/mycalamide A has been well reported as a powerful anticancer natural product based on eukaryotic protein synthesis inhibitors [52,56], a similar skeleton with a different side chain inspired the hybridization of the two natural products. It was anticipated that applying well-known biological properties of pederin to the psymberin-based would lead discovery [59] (Scheme 4).

Bishomoallylic alcohol **25**, a synthetic intermediate for total synthesis of psymberin [57], was transformed into tetrahydropyranyl amide **26** in eight steps, with a 29% yield. It lacks a unique dihydroisocoumarin chain in psymberin, while it possesses a dimethoxy group in pederin. In order to introduce a side chain of psymberin, acyl halide **27** was attached and reduced with NaBH_4_. Final deprotection using LiOH/MeOH condition afforded separable C_8_-diastereomeric mixture of **28** and **29**. These two psymberin-pederin chimeras were used to unveil the SAR of this natural product family. It is interesting to note that the absence of dihydroisocoumarin moiety gave rise to a significant loss of psymberin’s own cytotoxic property, while C_8_-epimer of the natural product caused a slight loss of this property. When they were treated to colon cancer cell line KM12, psymberin **25** or its C_8_-epimer showed a powerful or moderate toxicity (IC_50_ 0.45 nM and 37 nM, respectively). However, its truncated analog **28** or C_8_-epi analog **29** showed little or no toxicity to the same cell line (IC_50_ 710 nM and >1000 nM, respectively). These chimeras could be compared to pederin/mycalamide A as well. With a substituted tetrahydropyranyl side chain, which is rather common in natural pederin isomers, mycalamide A showed potent cytotoxicity (IC_50_ 0.95 nM) as well. It was proven that the common substituted side chain, or dihydroisocoumarin unit, plays a pivotal role in its own anticancer activity, employing a direct comparison of mother natural products and daughter chimeras. This case is a good indication of the effectiveness of a chimera strategy for drug development.

**Scheme 5:**
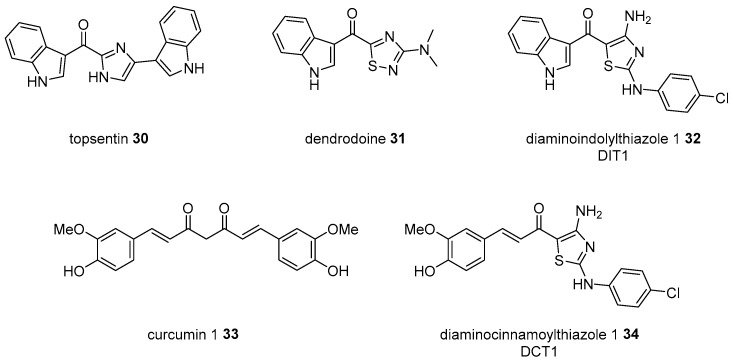
Hybridization from topsentin **30**, dendrodoine **31**, and curcumin 1 **33** [64].

Topsentin **30** is a bisindolyl marine alkaloid isolated from several marine sponges in Mediterranean *Topsentia genitrix* or Korean *Spongosorites* sp [60]. As this alkaloid exhibits excellent cytotoxicity to cancer cells and features a rather simple structure when compared to the other active marine natural products [61], numerous researchers have attempted to develop more potent and drug-like analogs [62]. One of the indole side chains in topsentin was changed to an aminothiazole unit, as a thiadiazole in dendrodoine **31**, another marine alkaloid from *Dendroda grossular* [63]. With the first chimera diaminoindolylthiazole (DIT) **32** in hand, additional hybridization with curcumin **33** was performed to yield diaminocinnamoylthiazole (DCT) **34** and its analogs [64] (Scheme 5). Moreover, DITs and DCTs were screened to the cancer cell line. It is interesting that DITs are very effective in the induction of apoptosis in HeLa cells (IC_50_ 1~45 μM), while DCTs play an active role in downregulating TNF-induced NF-κB activation. This research indicates that the hybridization of a simple functional group may also offer an opportunity to improve biological activity.

Neopeltolide **35** is another example of the chimera strategy of marine natural products. Since its isolation from marine sponge *Daedalopelta Sollas* in 2007 [65], urgent synthetic efforts were made to elucidate its correct structure [66]. As this marine macrolide showed various cytotoxic or cytostatic activity for numerous cancer cell lines, substantial research was conducted, including asymmetric synthesis, mechanism study, and analog synthesis. In addition, its antifungal activity was also focused upon. Contrary to its varied anticancer activity, its inhibitory activity to *Candida albicans* was highly potent at the minimum inhibitory concentration (MIC) 0.625 μg/mL [67]. This antifungal inhibition could be used for patients infected with the acquired immune deficiency syndrome (AIDS) or related fungal diseases. Further, the mechanism of action of neopeltolide was also studied to reveal that the agent works as the cytochrome bc_1_ complex inhibitor and inhibits the adenosine triphosphate (ATP) synthesis in mitochondria [68]. 

**Scheme 6:**
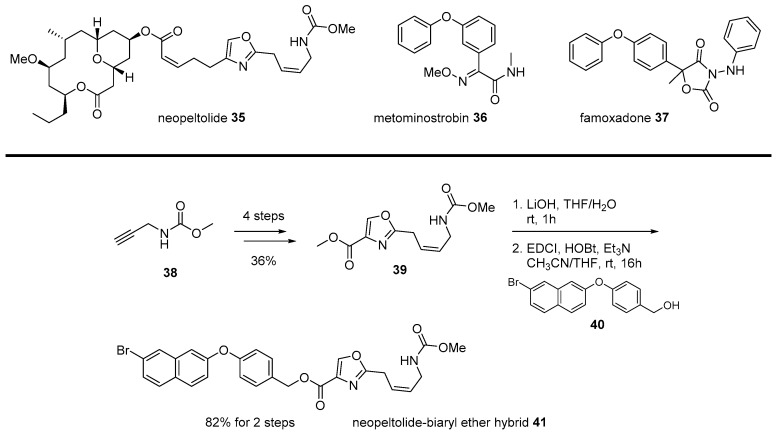
Hybridization from neopeltolide and biaryl ether hybrid [71].

Although systemic research on neopeltolide was undertaken, its application in drug discovery was hampered by its complex structure. Neopeltolide **35** features a cis-substituted tetrahydropyran skeleton with an ansacyclic macrolactone framework. This formidable structure made it difficult to prepare neopeltolide on a large scale or related analogs in a varied manner. An interesting strategy was the chimeric application of neopeltolide with biaryl ether active molecules [69]. An SAR study reported that the oxazolyl carbamate skeleton plays an important role in its activity profile, while a complex macrolide skeleton has little effect on it [70]. Instead of this complex and less important left chain in neopeltolide, simple biaryl moiety was inspired by other similar fungicide metominostrobin **36**, famoxadone **37**, or other biaryl ethers, as illustrated in Scheme 6 [71]. 

Methyl propargyl carbamate **38** was converted into oxazolyl ester **39** in four steps and with high yield. Ester **39** was hydrolyzed and esterified with various primary alcohol **40** with biaryl moiety. Among synthesized chimeras, naphthyl benzyl ether **41** showed the most potent inhibitory activity (IC_50_ 12 nM) for porcine succinate cytochrome c reductase (SCR). As this chimera simplified its mother structure, additional development for improved antifungal agent was expected.

**Figure 4:**
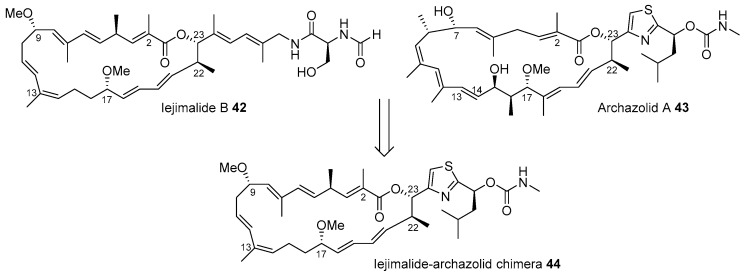
Design of iejimalide B and archazolid chimeras [72].

Figure 4 presents the hybridization of iejimalide and archazolid skeletons [72]. Iejimalide B **42** and its family compounds were isolated from *Eudistoma cf. rigida* or *Cystodytes* sp., which were collected from an island in Japan [73]. Due to its remarkable anticancer activity and selectivity to NCI 60 cell lines, this polyunsaturated macrolide has been focused upon for its promising role in drug discovery [74]. However, its scarcity in natural resources led to not only total synthesis [75,76] but also structural simplification based on a hybridization strategy [72]. Archazolid A **43**, isolated from terrestrial myxobacteria [77], possesses a very similar structure as that of iejimalide B **42;** however, archazolid A **43** features a relatively simple side chain with thiazole and methyl carbamate moiety. In addition, because archazolid **43** showed potent inhibitory activity to vacuolar-type ATPases as well [77], iejimalide-archazolid chimera **44** was designed and synthesized.

**Scheme 7:**
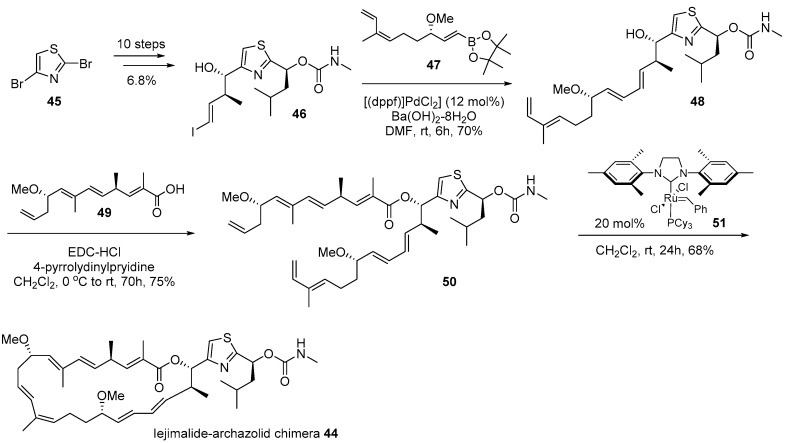
Synthesis of iejimalide-archazolid chimera **44** [72].

Dibromothiazole **45** was converted to vinyliodide **46** using a 10-step sequence containing the Corey-Bakshi-Shibata (CBS) reduction [78], Marshall alkylation [79], and lithium-halogen exchange reactions as shown in Scheme 7. It was coupled with known catechol borane [76] **47**, using the Suzuki coupling condition [80] to afford tetraene **48**. Finally, the esterification of tetraene **48** with known carboxylic acid [76] **49** and ring closing metathesis [81] produced the desired chimera **44** in good yield. Biological evaluation of chimera **44** against various cancer cell lines was performed and it was proven that this hybridization yielded reduced toxicity to cancer cell lines, although certain cell lines such as lung adeno (LXFA 629L, IC_50_ 0.32 μM) or colorectal (CSF HT29, IC_50_ 0.49 μM)) were slightly sensitive to chimera **44**.

### 2.2. Conjugation with Other Functional Compounds

For drug discovery and sequential development, marine natural products require more detailed studies, such as on-target protein isolation. The chemical conjugation of marine natural products with other functional compounds provides special opportunities in this regard. Biotin is a good example. Biotin, well known for its strong interaction with streptavidin [82,83,84], can be utilized to identify the target protein of natural products [85,86,87,88]. As this hybridization strategy may lead to the loss of its own binding affinity to natural products, it usually requires preliminary study to identify where to ligate biotin and what to use as a linker between biotin and the natural product. This pre-research requires additional efforts, thereby making it difficult to apply this method to a general natural product. However, in certain cases, well-designed chimeras showcase the usefulness of this hybridization protocol.

**Scheme 8:**
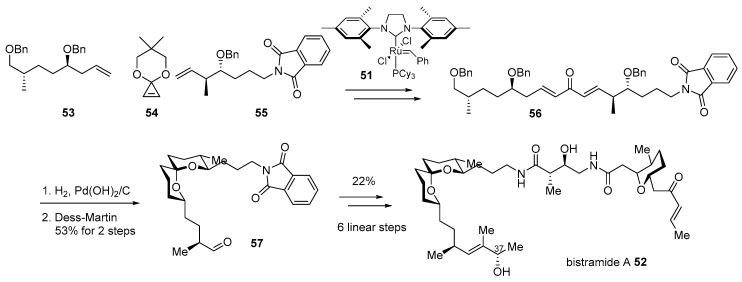
Total synthesis of bistramide A **52** [94].

Bistramide A **52**, isolated from marine metabolite of *Lissoclinum bistratum* [89,90,91], is a good example of this hybridization. As the bistramide family has shown potent cytotoxic properties on various cancer cell lines, such as non-small cell broncho-pulmonary carcinoma or HL60 cells as well as unique spiroketal skeleton [92], this marine product has been a captivating target for synthetic chemists. After pioneering research on structural elucidation and synthesis of skeletons or other isomers [93], enantioselective total synthesis of bistramide A was accomplished in 2004 [94]. This synthetic route features iterative cross metathesis of terminal alkenes **53** and **55** with the geometrically strained cyclopropene **54** [95] in the presence of Grubbs second-generation catalyst **55** to construct bisunsaturated ketone **56**. The treatment of H_2_ with Pd(OH)_2_/C to this ketone induced hydrogenation/hydrogenolysis and spontaneous cyclization to afford the desired spiroketal **57** in 53% yield after the Dess–Martin oxidation sequence. Ketal **57** was converted to bistramide A **52** after employing amidation with other building blocks in six linear steps. This efficient and convergent synthesis made it possible to not only elucidate the C_37_ chiral center [96] but also provide further opportunity for hybridization with other active molecules (Scheme 8).

**Figure 5:**
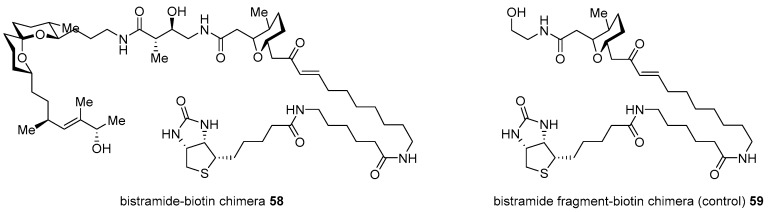
Biotin chimera based on bistramide A [98].

Although potent and active, bistramide A **52** has been reported since its first isolation [97]; its mechanism of action was unknown until the preparation of bistramide-biotin chimera, as illustrated in Scheme 8 [98]. Its protein kinase C(PKC) δ inhibitory features caused PKCδ to be a cellular target in HL-60 cells. However, an in vitro study with real-time fluorogenic kinase assay system [99] revealed that bistramide A did not have strong affinity to PKCδ as its inhibitory activity. In order to identify an early-stage target, target protein fishing was planned. Based on the synthetic strategy previously described [94], homologation with carbon linker and final attachment of biotin was accomplished in order to produce desired chimera **58** as well as fragment-biotin chimera **59** for a controlled experiment (Figure 5). The treatment of these two chimeras into whole-cell lysate from A549 cell revealed direct binding of monomeric G-actin (K*_d_* 7nM) as a primary target protein. Using this target fishing study with biotin-chimera enabled the study of a more detailed mechanism of action.

**Scheme 9:**
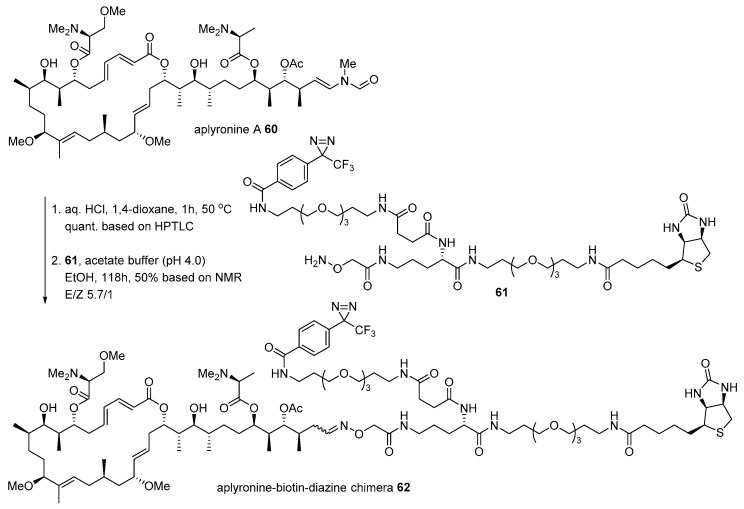
Preparation of aplyronine-biotin-diazine chimera **62** [101].

Aplyronine A **60**, isolated from Japanese sea hare *Aplysia kurodai* [100], is another case for the conjugation of marine natural product with biotin to identify the interaction of actin. While bistramide A **52** binds to actin, aplyronine A binds to the actin/tubulin complex, which is a 1:1:1 trimeric complex [101]. The Kigoshi group treated aplyronine A with aqueous HCl to produce the corresponding aldehyde, which was condensed with hydroxylamine **61** to form an oxime bond as an inseparable mixture of aplyronine-biotin-diazine chimera **62** (Scheme 9). When it was treated to HeLa S3 cells, α/β tubulins appeared in SDS-PAGE with some other nonspecific binding proteins. In order to validate this Western blot result, purified actin (from rabbit) or tubulin (from porcine brain) was treated with chimera **62**. This validation study examined that unusual aplyronine/actin/tubulin heterotrimeric complex is responsible for its extraordinary antitumor effect through cell arrest at mitosis in HeLa S3 cells (IC_50_ 0.45 nM) [102]. 

**Figure 6:**
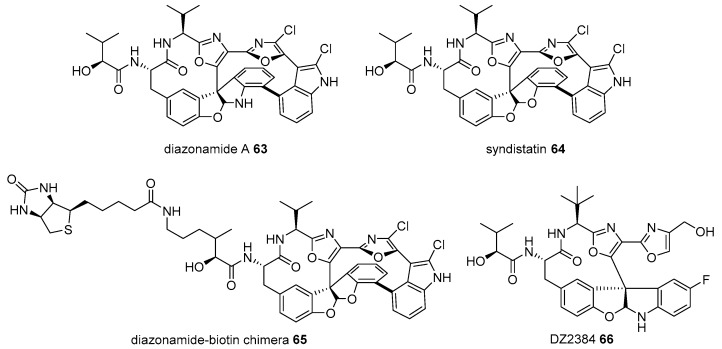
Preparation of biotinylated diazonamide and its advanced analog DZ2384 **66** [107].

Diazonamide A **63** is a marine natural product from *Diazona chinensis*, located on the ceilings of small caves on the Siquijor Islands in the Philippines [103]. Despite its scarcity in natural sources (54 mg from 256 g of *D. chinensis*), its extraordinary antimitotic property in human colon carcinoma and murine melanoma cancer cell lines has garnered substantial interest from chemists and biologists [104,105,106]. This tremendous study led to structural elucidation, efficient chemical synthesis, and related analog preparation. Its target protein study is interesting. When diazonamide (syndistatin **64**)-biotin chimera **65** was prepared and treated with HeLa extract, two additional bands of an affinity matrix were observed on an SDS/PAGE compared to those in the control experiment. Mass identification indicated that these two bands were ornithine δ-amino transferase, although why the matrix separates is unknown [107]. As ornithine δ-amino transferase is well known as a mitochondrial enzyme in the TCA cycle [108], it was slightly surprising. However, further study using the RNA*i*-mediated knockdown method and additional investigation proved that ornithine δ-amino transferase is essential for mitotic cell division. Thus, target protein and its corresponding pathways of diazonamide were unveiled to lead to the discovery of advanced diazonamide analog DZ2384 **66 [109]**. This process shows a 5–50 times higher efficacy without neurotoxicity at an effective dose (Figure 6).

**Scheme 10:**
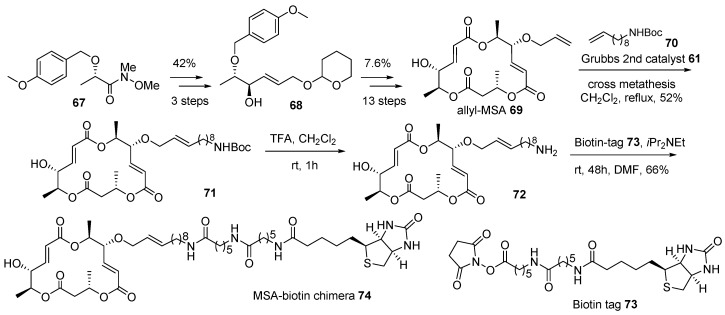
Preparation of biotinylated marcosphelide chimera **74** [110].

Further, macrosphelide (MS) skeleton was also utilized in this chimeric research, as depicted in Scheme 10 [110]. Although it has a structural framework and biological profiles, its mechanism of action is not well studied in the MS family. In order to identify the target protein of MSA **2** and elucidate the following pathway, MSA-biotin hybridization was planned. The protected Weinreb-amide of (*S*)-lactic acid **67 [111]** was transformed into allylic alcohol **68** using a three-step protocol—alkylnylatoin, carbonyl reduction, and alkyne reduction—in 42% yield. This key intermediate **68** in hand, allylation, iterative esterification, and deprotection sequence finally afforded allyl-MSA **69** in 7.6% yield and 13 steps. Biotin was linked to allyl-MSA **69** after additional manipulation. For efficient target fishing, a long carbon chain was selected as a linker. Terminal alkene in allyl-MSA was utilized in cross metathesis to introduce a long carbon linker and protect the amine functional group in amino MSA **71**. Finally, acidic deprotection and following amidation with biotin tag **73** produced the desired MSA-biotin chimera **74**. Target fishing studies, employing this chimera, on potent cell-cell adhesion inhibitor are ongoing.

**Scheme 11:**
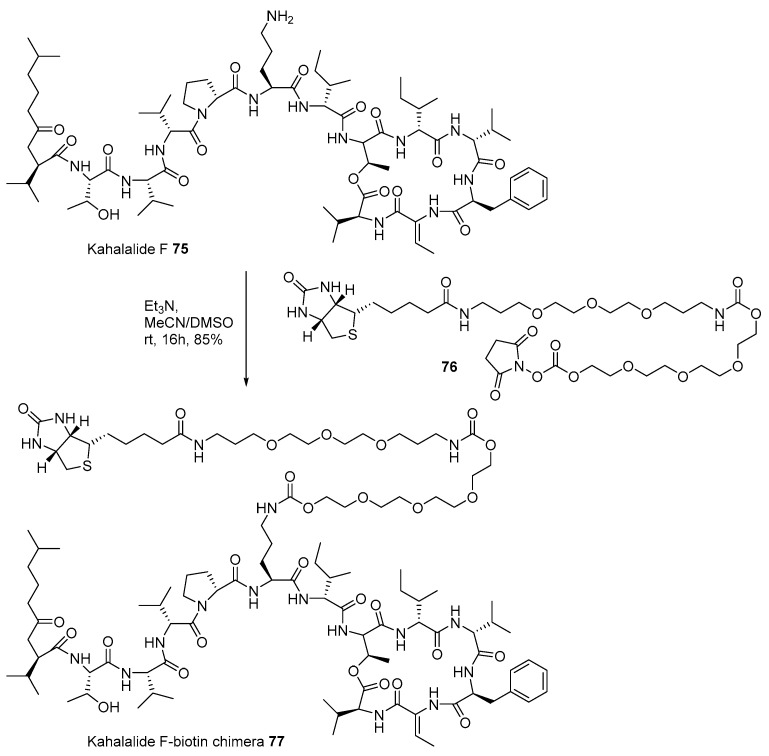
Preparation of Kahalalide F-biotin chimera **77** [114].

The marine natural product-biotin chimera strategy was also applied to identify the target protein of kahalalide F **75**. Ever since the isolation of kahalalide F from marine mollusk, *Elysia rufescens*, in 1993 [112], this polypeptide marine product has been used in clinical trials because of its powerful anticancer activity and low cellular toxicity [113]. For advanced development, identification of the target protein using biotinylation of kahalalide F was also attempted, as presented in Scheme 11. Kahalalide F was coupled with biotinylated linker **76 [114]**, prepared from biotin and tetraethyleneglycol, under a weak basic condition to produce desired chimera **77** in 85% yield. When chimera 77 was treated to T7 cDNA phage for reverse chemical proteomics, human ribosomal protein S25 was found to be responsible for its potent anticancer property in a dose-dependent manner.

**Scheme 12:**
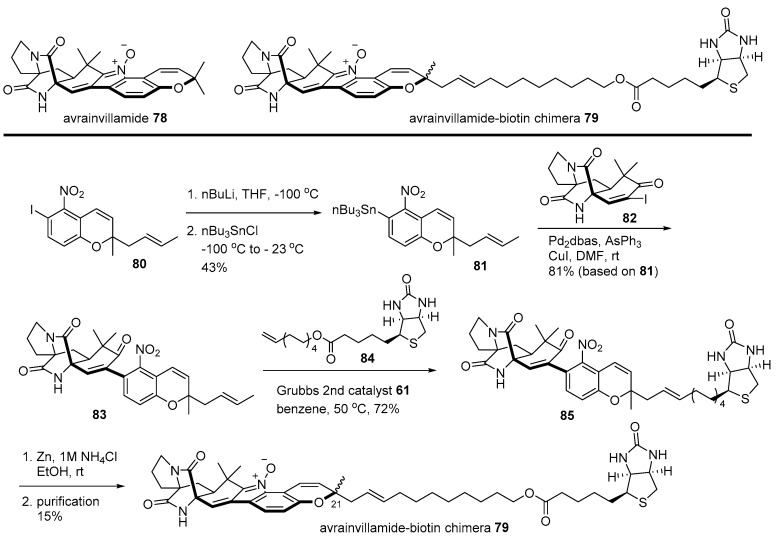
Preparation of avrainvillamide-biotin chimera **79** [115].

Avrainvillamide is another good example of the biotin chimera strategy [115]. In order to unveil the mysterious target protein of powerful avrainvillamide, isolated from *Aspergillus* sp. CNC358 [116], a biotinylated natural product **79** was designed. Synthesis of chimera **79** is depicted in Scheme 12. Iodoarene **80** [116] was stannylated to yield aromatic stannane **81** via metal/halogen exchange and a substitution reaction. Then, stannane **81** was coupled with vinyl iodide **82** [117] to produce nitroarene **83**, which was converted into biotinylated arene **85** employing cross metathesis with terminal alkene **84 [118]**. Reductive cyclization of **85** and HPLC purification afforded the desired chimera **79** (1:1 mixture of diastereomers at the C21 chiral center). Finally, Western blotting and MS/MS sequencing of these chimera-treated T-47 D cells revealed that nucleophosmin was a target protein of avrainvillamide. As nucleophosmin is a multifunctional protein in numerous tumors [119], a more detailed study was subsequently conducted to disclose the mode of action of avrainvillamide and its related natural product stephacidin [120]. 

**Scheme 13:**
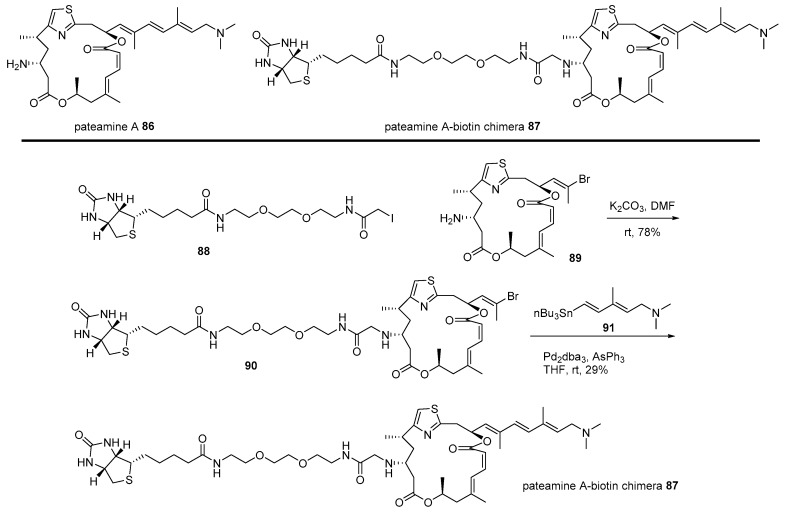
Preparation of pateamine A-biotin chimera **87** [124].

The hybridization strategy was also utilized to identify the target protein of pateamine, which is isolated from the New Zealand marine sponge [121]. As this marine natural product showed not only remarkable cytotoxicity to tumor cell line P338 [121] but also extraordinary immunosuppressive activity [122], target protein identification was pursued, as illustrated in Scheme 13 [123]. Primary iodide **88** [124] was alkylated with truncated pateamine **89** [123], which is more stable than pateamine **86**. Further, corresponding vinyl bromide **90** [124] was coupled with vinyl stannane **91** [122] to produce the desired pateamine-biotin chimera **87** in moderate yield. The pull-down method of this affinity-matrix **87** showed that it bound to and inhibited the association of eIF4A and eIF4B, finally inhibiting the cap-dependent eukaryotic translation sequence [124]. This fundamental study has also opened the way for more detailed research and further applications of pateamine analogs [125]. 

## 3. Conclusions

As an endless resource of bioactive molecules, marine natural products stand a reliable chance in the world of drug discovery. However, certain limitations of these products, such as complex structure, toxicity, selectivity, and even potency have driven researchers to identify additional strategies for these natural compounds. Chimeras yielded by the hybridization of two active molecules are one such solution. Changing the active skeleton of the mother framework also added more opportunities, but this process also comes with its own set of unique drawbacks, such as loss of function, scarcity of supply, and complex structure for chemical modification. In order to overcome these limitations, SAR, structural simplification, development of the synthetic route, and the medicinal chemistry approach have been studied thus far. Based on this fundamental progress, currently, hybridization of active natural products is being utilized to improve their own biological properties and elucidate veiled mechanism of actions. The efficient application of this chimera strategy to other active molecules remains a direction for groundbreaking research in the future.
